# Drug discovery from Chinese medicine against neurodegeneration in Alzheimer's and vascular dementia

**DOI:** 10.1186/1749-8546-6-15

**Published:** 2011-04-22

**Authors:** Yuen-Shan Ho, Kwok-Fai So, Raymond Chuen-Chung Chang

**Affiliations:** 1 Laboratory of Neurodegenerative Diseases, Department of Anatomy, The University of Hong Kong Pokfulam, Hong Kong SAR, China; 2 Research Centre of Heart, Brain, Hormone and Healthy Aging, LKS Faculty of Medicine, The University of Hong Kong Pokfulam, Hong Kong SAR, China; 3 State Key Laboratory of Brain and Cognitive Sciences, The University of Hong Kong Pokfulam, Hong Kong SAR, China

## Abstract

Alzheimer's disease and vascular dementia are two major diseases associated with dementia, which is common among the elderly. While the etiology of dementia is multi-factorial and complex, neurodegeneration may be the major cause of these two diseases. Effective drugs for treating dementia are still to be discovered. Current western pharmacological approaches against neurodegeneration in dementia develop symptom-relieving and disease-modifying drugs. Current integrative and holistic approaches of Chinese medicine to discovering drugs for neurodegeneration in dementia include (1) single molecules from the herbs, (2) standardized extracts from a single herb, and (3) herbal formula with definite composition. This article not only reviews the concept of dementia in western medicine and Chinese medicine but also evaluates the advantages and disadvantages of these approaches.

## Introduction

Alzheimer's disease (AD) and vascular dementia (VaD) are the major forms of dementia. In addition, in the postmortem brains of the late stage of Parkinson's disease/Lewy body disease also find pathological hallmarks of AD [1]. Senile dementia is the progressive decline of memory and some related cognitive functions in the elderly. The global dementia population is predicted to reach 81.1 million by 2040 [2]. In 2010, the estimated prevalence of senile dementia in China is 6.0 to 7.0 million, accounting for about one-sixth of the global prevalence; the prevalence is expected to increase to 22.5 million by 2040, accounting for one-fourth of the global prevalence by that time [3]. The rapid increase in the number of dementia patients urgently demands effective prevention and treatment. Current approaches to dementia-related neurodegenerative diseases still highly rely on relieving symptoms. As some Chinese medicinal herbs have been used in treating dementia, many researchers are now turning to Chinese medicine for identifying potential neuroprotective agents or disease-modifying agent. This article reviews the strategy in the research of Chinese medicine in dementia related-neurodegenerative diseases.

### Dementia and medical sciences

AD is clinically characterized by the progressive loss of memory, cognitive functions and behavioral changes. The pathogenesis of AD has been widely studied [4,5], in which beta-amyloid (Aβ) peptide and hyperphosphorylated tau protein as components of extracellular senile plaques and intracellular neurofibrillary tangles, respectively, are believed to be the targets for developing disease-modifying drugs. Current AD treatments are all symptom-relieving agents and heavily rely on the use of acetylcholinesterase (AChE) inhibitors (donepezil, rivastigmine and galantamine). AChE inhibitors slow down the degradation of the neurotransmitter acetylcholine, thereby increasing its bioavailability. Another approved AD treatment aims to reduce glutamate excitotoxicity. Memantine, the only approved drug in this category, acts as a non-competitive *N*-methyl-D-aspartate (NMDA) receptor antagonist to reduce glutamate-mediated neurotoxicity [6].

Development and progression of VaD are associated with a number of risk factors, many of which are related to the pathogenesis of atherosclerosis [7]. Stroke is also a critical factor for VaD; it was reported that 79.5% of VaD patients had a history of stroke [8]. As there is no cure for VaD, management of VaD emphasizes on the prevention of new stroke and control of vascular risk factors.

### Dementia and Chinese medicine

According to Chinese medicine theory, there is no distinction between AD and VaD. Dementia is caused by (1) deficiency of vital energy of the Kidney (*Shen*), Marrow (*Sui*), Heart (*Xin*) and Spleen (*Pi*) and (2) stagnation of Blood (*Xie*) and/or Phlegm (*Tan*). Thus, herbs used for dementia are not specific for the nervous system but tend to be multi-functional [9].

#### Standardization of dementia subtype classification and research guidelines

*Guideline for Chinese Medicine Diagnosis, Classification and Clinical Research of Senile Dementia *was published in 1990. The guideline classified dementia into six subtypes according to the CM theory: (1) the Bone Marrow (*Gusui*) deficiency syndrome, (2) the Liver (*Gan*) and Kidney (*Shen*) Yin deficiency syndrome, (3) the Spleen (*Pi*) and Kidney (*Shen*) Yang deficiency syndrome, (4) the Qi stagnation and Blood (*Xie*) stasis syndrome, (5) the Phlegm Turbid (*Tan Zhuo*) blocking Orifice (*Qing Qiao*) syndrome, and (6) the Heart (*Xin*) and Liver Fire (*Gan Huo*) syndrome [10]. Since then, clinical studies on dementia in China have been based on this guideline [11]. More recently, the *Guideline Principles for Clinical Research on New Chinese Medicine *(trial version) [3] provides more detailed description on the diagnostic criteria and describes the severity of disease subtypes quantitatively. The Mini-Mental State Examination (MMSE) score has also been introduced as the main reference index [3]. *Criteria for the Diagnosis, Differentiation of Syndrome and Evaluation of Efficacy of Vascular Dementia for Research Studies *were published on 2002, emphasizing that the diagnosis of VaD must meet the NINDS-AIREN criteria (developed by the National Institute of Neurological Disorders and Stroke (NINDS) and the Association Internationale pour la Recherche et l'Enseignement en Neurosciences (AIREN)) and that the differentiation of syndromes in Chinese medicine should be based on the scale for the differentiation of syndromes of vascular dementia (SDSVD) published in 2000 [12]. It classifies VaD in 7 syndromes according to CM diagnosis: (1) the Kidney Essence (*Shen Jing*) deficiency syndrome, (2) the Phlegm Turbid (*Tan Zhuo*) blocking Orifice (*Qing Qiao*) syndrome, (3) the vessels obstructed by Blood Stasis (*Xie Yu*) syndrome, (4) the brain aggressed by Liver's (*Gan*) *Yang *syndrome, (5) the Heat (*Re*) and Toxin (*Du*) accumulation syndrome, (6) the Qi and Blood (*Xie*) deficiency syndrome, and (7) the constipation and toxin in intestines syndrome. SDSVD employs a detailed scoring system to assist syndrome differentiation and diagnosis [13].

#### Chinese medicine approaches based on the etiology of dementia

Chinese medicine theory considers dementia to be a more holistic and integrated approach, rather than a problem in just one organ. Dementia is complex and may involve multiple causes. During progression of dementia, the significant of different pathological factors may also change. In Chinese medicine, it is believed that the disease is highly correlated to the abnormal functions of other organs including the Kidney (*Shen*), Liver (*Gan*), Heart (*Xin*) and Spleen (*Pi*), although the pathological site of dementia is in the brain. For example, dementia patients who initially have Kidney (*Shen*) deficiency may also develop stagnation of Blood (*Xie*) and Phlegm (*Tan*) leading to dementia. All these clinical experiences, stagnation of blood and kidney deficiency, become two important concepts in Chinese medicine to explain the origin of sickness leading to dementia [14-16].

#### Chinese medicine studies on the prevalence and distribution of dementia subtypes

In order to integrate Chinese medicine diagnosis, some researchers investigated the prevalence and distribution of dementia subtypes. Wang *et al. *found that deficiency of Qi, Blood (*Xie*), Essence (*Jing*) was present in most dementia cases and that Heart (*Xin*) and Kidney (*Shen*) were most commonly afflicted by the condition [17]. Yang *et al. *found that stagnation of Blood (*Xie*) and Phlegm (*Tan*) were frequently present in moderate and severe AD cases [18]. While these data are not diagnostic criteria, they provide important information for the prevention of pathological progression in dementia.

### Chinese medicine research on treatment of dementia

Nowadays, the development of Chinese herbal medicine mainly adopted three approaches, which include the single molecule approach, standardized extracted approach and fixed herbal formula approach. In the following sections, we will discuss the recent development of each approach. It is beyond the scope of our report to review the pharmacological effects of all medicinal herbs for dementia treatment in detail. In fact, our aim is to use several representative examples to illustrate the advantages and disadvantages of each approach.

#### Single molecules from a single herb

##### Huperzine A

Huperzine A is an alkaloid isolated from *Huperzia serrata *(*Qiancengta*) which is documented in Chinese medicine literature as an anti-inflammatory herb for relieving pain and alleviating swelling after trauma. According to the Chinese medicine theory, *Huperzia serrata *helps removing Heat (*Re*) and has detoxification effects. Huperzine A is widely used in China to treat AD. Clinical trials demonstrated that huperzine A significantly improved cognitive functions in AD patients [19] and its potential therapeutic effects for VaD [20]. While evidence for the use of huperzine A to treat AD was insufficient, a recent Cochrane systematic review reported no obvious adverse effects in AD patients treated with huperzine A [21].

The anti-AChE activity of huperzine A is the basis for its use to treat dementia patients [22]. Animal studies found that huperzine A was comparable to donepezil and rivastigmine in terms of anti-AChE activity [23]. *In vivo *studies showed that huperzine A affected amyloid precursor protein processing to reduce the formation of Aβ peptides [24]. Other studies also showed that huperzine A attenuated apoptosis in neurons treated with Aβ peptides [25]; and huperzine A elicited anti-oxidative effects which allowed it to protect neurons against hydrogen peroxide and Aβ-induced oxidative damages [26,27]. Huperzine A was found to improve mitochondrial functions in neurons and reduce the level of reactive oxygen species in neurons exposed to Aβ peptides [28].

##### Pros and cons of the single molecule approach

Huperzine A is a chemical drug derived from Chinese medicine as a chemical library [29]. The discovery of huperzine A as an anti-AChE inhibitor was based on the observation that the administration of *Huperzia serrata *extract induced cholinergic stimulation in schizophrenic patients [30]. As the quality and bioavailability of a herb can be affected by the cultivation environment and harvesting season [31], single molecule approach eliminates or minimizes this variation.

However, this approach may also generate problems. Extraction of active ingredients is often not a simple task. Interactions of ingredients during preparation procedure are essential to the therapy. Moreover, evidence shows that single component extracted from plants is less potent than crude extract [32]. Researchers often do not use any Chinese medicine theory as the basis for their investigation when studying these compounds. Therefore, some Chinese medicine experts are skeptical about the approach [33].

#### Standardized extracts from a single herb

##### Authentication of herbs

Historically, herbs grown in a particular habitat are considered *Daodi *(genuine) [34]. Today, the good agricultural practice (GAP) promoted in China ensures the quality and consistency of a particular herb [35]. A herbal extract is considered 'standardized' if (1) the raw material (herb) is grown and collected according to the GAP; (2) the extraction follows a well-defined procedure; and (3) the chemical profiles are consistent among batches of extracts.

##### EGb761

EGb761 is a standardized herbal extract from the dried leafs of *Ginkgo biloba*, containing approximately 24% flavone glycosides (quercetin, kaempferol and isorhamnetin) and 6% terpene lactones (ginkgolides A, B, C, J and bilobalide) [36]. Dried fruit of *Ginkgo biloba *is used in Chinese medicine to treat asthma and coughing. While the chemical and biological properties of individual EGb761 component have been investigated *in vitro *and *in vivo *[37-39], the standardized extract EGb761 is often used in clinical research [40]. A Cochrane systematic review did not support the use of EGb761 in dementia treatment [40]. Another report also suggested that EGb761 was not effective in reducing the incidence of AD [41]. Although clinical efficacy of EGb761 for dementia treatment is still controversial, the use of a well-defined herbal extract in clinical studies has been demonstrated.

##### Chinese medicine theories and anti-dementia drug research

Chinese medicine theory and western pharmacology may be integrated for the development of anti-dementia Chinese herbal extracts. According to Chinese medicine theory, the fruit of *L. barbarum *(*Gouqizi*), which is used to tonify the Yin in our body, nourishes our Eye (*Yan*), Liver (*Gan*) and Kidney (*Shen*); its anti-aging effects are well-documented in Chinese medicine literature [42]. In our laboratory, research of standardized *L. barbarum *extract is based on Chinese medicine concepts. Firstly, *L. barbarum *is chosen as the research candidate because of its unique Chinese medicine properties. Secondly, the research direction of our standardized *L. barbarum *extract was inspired by Chinese medicine theory. Owing to anti-aging properties, *L. barbarum *may alleviate aging-associated neurodegenerative diseases such as AD and VaD [9]. Quality control of our raw materials ensured the quality of our *L. barbarum *extract [43]. We found that *L. barbarum *extract attenuated Aβ peptide induced neuronal apoptosis [43]. The holistic concept in Chinese medicine inspired us to study the effects of the extract on other dementia related pathological and risk factors. We then discovered that *L. barbarum *extract protected neurons against glutamate toxicity, suggesting that it might slow down dementia progression [44]. We also demonstrated that *L. barbarum *extract protected neurons against homocysteine toxicity where hyperhomocysteinaemia is a risk factor for AD [45].

#### Herbal formulations

##### Yokukansan

*Yokukansan*, or TJ-54, is a Kampo herbal remedy originating from the Chinese herbal formula *Yigan San *developed in the Song Dynasty for the treatment of Liver (*Gan*) dysfunction-induced agitation and restlessness in children. *Yigan San *consists of seven herbs, namely *Angelica acutiloba *(Danggui), *Atractylodes lancea *(*Baishu*), *Bupleurum falcatum *(*Chaihu*), *Poria cocos *(*Fuling*), *Cnidium officinale *(*Chuanxiong*), *Uncaria rhynchophylla (Gouteng) *and *Glycyrrhiza uralensis *(*Gancao*) at a ratio of 3:4:2:4:3:3:1.5. This composition is also used in *Yokukansan *[46]. Since this remedy is used for the treatment of psychiatric disorder, the possible therapeutic effects on dementia symptoms are under investigation.

Both clinical and preclinical studies on *Yokukansan *support its use in dementia treatment. A randomized, observer-blind, controlled trial found that a 4-week *Yigan San *treatment improved the behavioral and psychological symptoms of dementia (BPSD) [47] which includes aggression, agitation, screaming, wandering, hallucinations and delusions. These symptoms develop in 20-80% of dementia patients at different stages [48]. *Yigan San *reduced cholinesterase inhibitor-resistant visual hallucination in a small group of patients of dementia with Lewy bodies [49]. Positive effects of *Yokukansan *on sleep disturbance in dementia patients were also reported [50]. A randomized cross-over study (subjects receiving active treatment or placebo in different stages of the trial) found that *Yokukansan *significantly improved the BPSD in AD patients but had no effects on their cognitive functions as demonstrated by the MMSE score. Effects of *Yokukansan *could persist for one month and was well-tolerated [51]. Other studies also found that *Yokukansan *was safe and effective in treating BPSD in AD and even PD patients [52,53]. *Yokukansan *might modulate the glutamatergic neurotransmitter system; hence protecting neurons against excitotoxicity [54,55]. *Yokukansan *provided direct protection on neurons or through modulating the glutamate reuptake by astrocytes [56]. *Yokukansan *also affected the expression of serotonin receptor in the frontal cortex of mice injected with 2,5-dimethoxy-4-iodoamphetamine [57].

##### Challenges in developing anti-dementia herbal formulations

Apart from *Yigan San*, some other Chinese herbal formulae are effective in treating the dementia [58,59]. Most of these studies are single clinical trials performed on a single formula. There are few studies on action mechanisms. Moreover, few studies use Chinese medicine diagnostic criteria. In other words, the effects of a definite formula were tested on patients regardless of their dementia subtype in Chinese medicine diagnosis. As exemplified in a clinical trial for respiratory diseases, it should be feasible to incorporate Chinese medicine diagnosis in the clinical trials for dementia [60].

## Conclusion

While Chinese herbal medicine is considered a big chemical library, potential drugs of single molecules have been developed for the treatment of AD and VaD but Chinese medicine concepts have not been fully incorporated for new drug development in Chinese medicine. As Chinese medicine aims to restore harmony of the whole body rather than only target the brain in treating encephalopathy [61], further research into experimental and clinical sciences should be conducted to explain how Chinese medicine can treat and prevent AD and VaD.

## Abbreviations

AD: Alzheimer's disease; Aβ: beta-amyloid; AChE: acetylcholinesterase; BPSD: behavioral and psychological symptoms of dementia; MMSE: Mini-Mental State Examination; NINDS-AIREN: National Institute of Neurological Disorders and Stroke (NINDS) and the Association Internationale pour la Recherche et l'Enseignement en Neurosciences (AIREN); SDSVD: scale for the differentiation of syndromes of vascular dementia; VaD: vascular dementia.

## Competing interests

The authors declare that they have no competing interests.

## Authors' contributions

KFS and RCCC contributed the main theme ideas. YSH wrote the manuscript. All authors read and approved the final version of the manuscript.
